# Supplementary material for: Genetic diversity increases with depth in red gorgonian populations of the Mediterranean Sea and the Atlantic Ocean
Source: PeerJ. 2019 May 24;7:e6794. doi: 10.7717/peerj.6794 (PMC6536111; doi:10.7717/peerj.6794)
Supplement: S4 [file peerj-07-6794-s004.docx]

**Genetic diversity increases with depth in red gorgonian populations of the Mediterranean Sea and the Atlantic Ocean**

Joanna Pilczynska, Silvia Cocito, Joana R. H. Boavida, Ester A. Serrão, Jorge M. Assis, Eliza Fragkopoulou, Henrique Queiroga

**S4. Comparison of deep and shallow bottom temperatures using high-resolution environmental data on a daily basis.**

**Methods**

To characterize the thermal environment and assess differences between depth levels, information on ocean temperatures was compiled on a daily basis for a 20-year period (1993-2013). Surface temperature data derived from the Operational Sea Surface Temperature and Sea Ice Analysis, a dataset that combines microwave and infrared satellite data with in situ measurements, on a spatial resolution of ~5km (Stark et al., 2011). Temperatures at 20, 30 and 60m depth derived from the Hybrid Coordinate Ocean Model (HYCOM), a product forced by heat flux, wind speed, wind stress and precipitation on a spatial resolution of ~7km. The HYCOM model can resolve complex oceanic processes such as eddies, meandering currents, filaments and fronts (Chassignet et al., 2007) and accurately predict temperature temporal variation with low averaged bias ranging from 0.2°C to 0.3 °C (Kara et al., 2008; 2010). For each sampling site and depth level, temperature estimates were produced with trilinear interpolation (Assis et al., 2017).

The number of consecutive days with temperature above 25°C (physiological lethal temperature for *Paramuricea clavata*; Boavida et al., 2016) was determined for the Mediterranean Sea. For the Atlantic Ocean we estimated the number of consecutive days with temperature above 20°C, because water temperature at the Atlantic sites never reached 25°C during the studied period and depths.


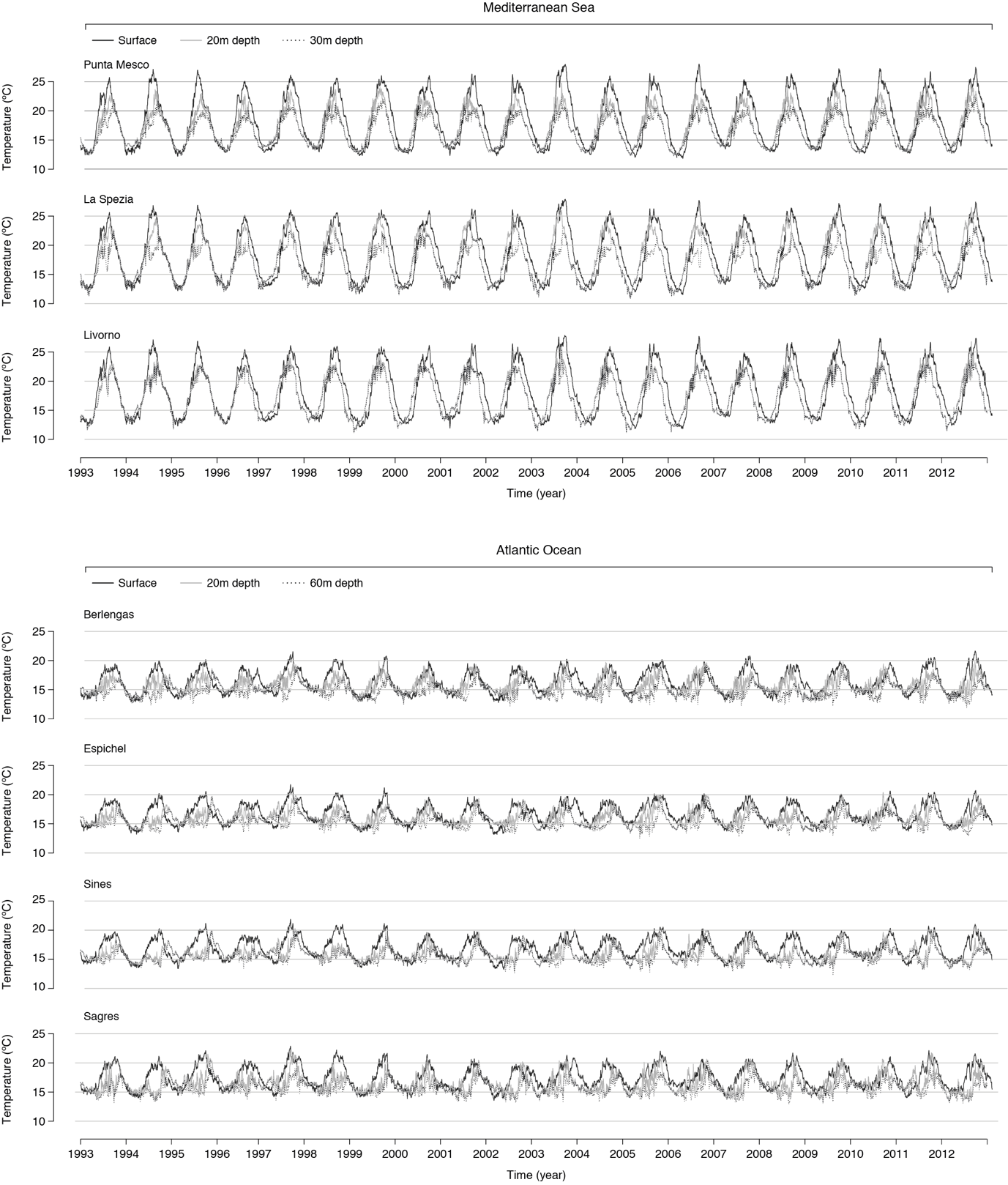


Fig 1. Temperature time series at exemplar Mediterranean and Atlantic sites at different depths. Surface waters are warmer when compared to deeper ones, and with a wider amplitude of observed temperatures (deeper waters tend to be more stable). The Mediterranean Sea has higher maximum values both in shallow and deeper waters when compared to the Atlantic.


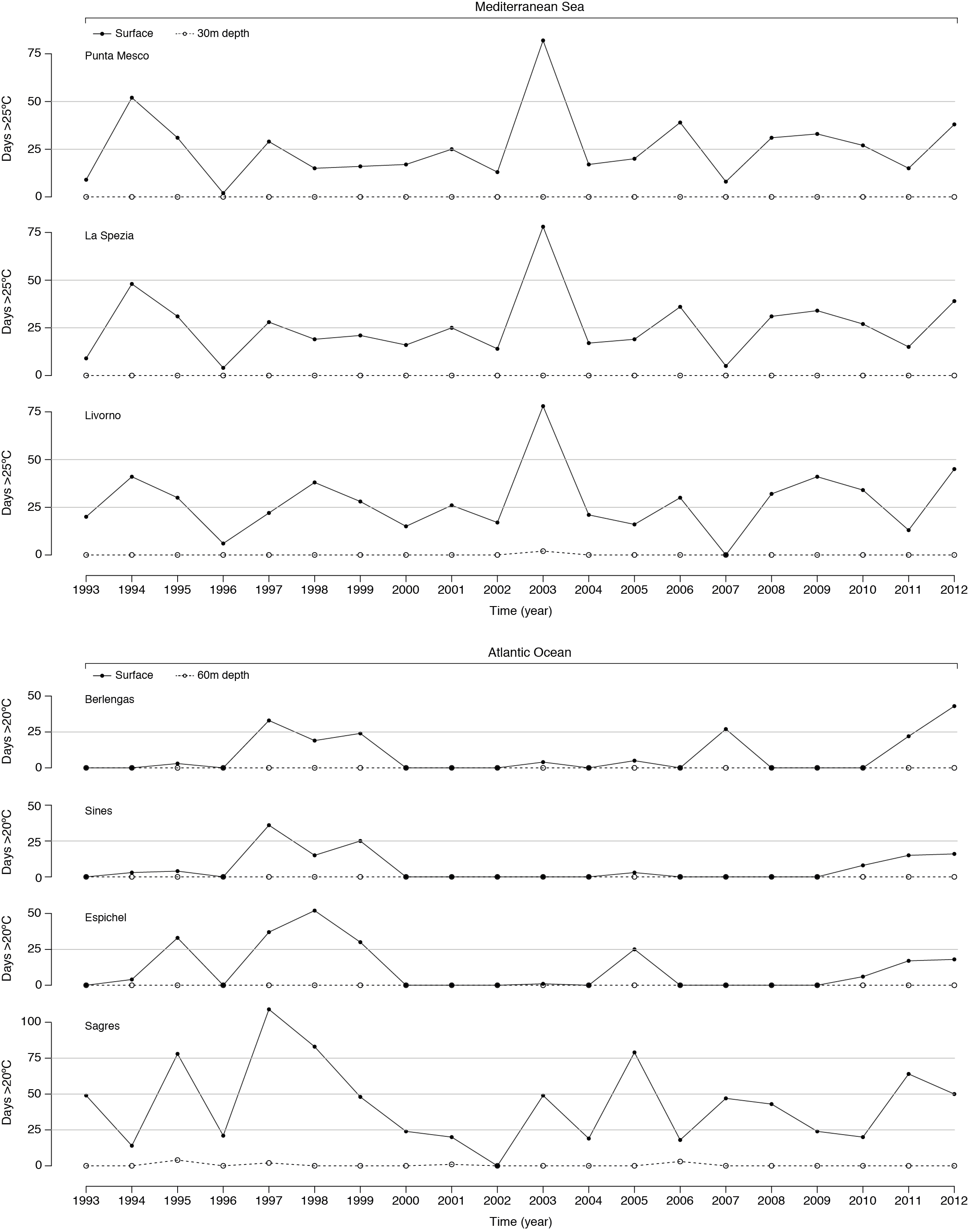


Fig 2. Number of consecutive days with temperatures above 25°C in exemplar Mediterranean sites (top panel) and above 20°C in Atlantic sites (lower panel). Surface and 30m deep temperatures are depicted by a continuous and a dashed line, respectively.

Table 1. Mean and range of temperatures for exemplar sites and depth levels (period 1993-2003). 60m and 30m represent the maximum sampling depth in the Atlantic and Mediterranean, respectively.

| **Site** | **surface** | | | | **20m** | | | | **60m; 30m** | | | |
| --- | --- | --- | --- | --- | --- | --- | --- | --- | --- | --- | --- | --- |
|  | min | max | mean | SD | min | max | mean | SD | min | max | mean | SD |
| **Berlengas** | 12.78 | 21.66 | 16.39 | 1.91 | 12.56 | 19.98 | 15.74 | 1.38 | 11.89 | 19.18 | 14.82 | 1.17 |
| **Espichel** | 13.17 | 21.74 | 16.72 | 1.72 | 13.46 | 20.51 | 16.08 | 1.29 | 12.51 | 19.79 | 15.33 | 1.15 |
| **Sines** | 13.26 | 21.87 | 16.86 | 1.76 | 12.89 | 20.29 | 15.92 | 1.26 | 12.35 | 19.94 | 15.47 | 1.17 |
| **Sagres** | 13.55 | 22.89 | 17.46 | 1.91 | 13.05 | 21.76 | 16.47 | 1.50 | 12.91 | 20.59 | 15.64 | 1.17 |
| **Punta Mesco** | 11.83 | 28.03 | 18.41 | 4.38 | 12.28 | 24.85 | 17.23 | 3.13 | 12.27 | 22.51 | 16.50 | 2.45 |
| **La Spezia** | 11.51 | 27.89 | 18.40 | 4.42 | 10.93 | 26.50 | 17.70 | 3.83 | 10.90 | 23.44 | 16.72 | 2.84 |
| **Livorno** | 11.82 | 27.84 | 18.45 | 4.36 | 11.12 | 26.79 | 17.55 | 3.52 | 11.18 | 25.46 | 17.32 | 3.32 |

For two decades (1993-2013), temperatures were higher in the Mediterranean Sea compared to the Atlantic Ocean. Mediterranean Sea temperature ranged from 12 to 28ºC in shallow and from 11 to 25ºC in deep sites, frequently reaching over 25ºC for approximately 25 days per year in the shallow waters (Fig. 3; Table S1; Fig. S2). In the Atlantic Ocean, sea temperature ranged from 13 to 23ºC in shallow and 12 to 21ºC in deep sites, reaching over 20ºC for approximately 25 to 50 consecutive days in shallow waters, during especially warm summers (Fig. 3; Table S1; Fig. S2).

**References**

Assis J, Tyberghein L, Bosch S, Verbruggen H, Serrão EA, De Clerck O. (2017). Bio-ORACLE v2.0: Extending marine data layers for bioclimatic modelling. *Global Ecology and Biogeography*, 27, 277–284.

﻿Boavida, J., Assis, J., Silva, I., & Serrão, E. A. (2016). Overlooked habitat of a vulnerable gorgonian revealed in the Mediterranean and Eastern Atlantic by ecological niche modelling. Scientific Reports, 6(October), 36460.

Chassignet EP, Hurlburt HE, Smedstad OM, Halliwell GR, Hogan PJ, Wallcraft AJ, Baraille R, Bleck R. (2007). The HYCOM (hybrid coordinate ocean model) data assimilative system. *Journal of Marine Systems*, 65, 60-83.

Kara AB, Metzger EJ, Hurlburt HE, Wallcraft AJ, Chassignet EP. (2008). Multistatistics metric evaluation of ocean general circulation model sea surface temperature: Application to 0.08 Pacific Hybrid Coordinate Ocean Model simulations. *Journal of Geophysical Research: Oceans*, 113.

Kara AB, Hurlburt HE, Barron CN, Wallcraft AJ, Metzger EJ. (2010). Can an atmospherically forced ocean model accurately simulate sea surface temperature during ENSO events? *Tellus A*, 62, 48-61.

Stark C, Breitkreutz BJ, Chatr-Aryamontri A, Boucher L, Oughtred R, Livstone MS, et al. (2011). The BioGRID interaction database: 2011 update. *Nucleic Acids Research*, 39, 698-704.
